# Supplementary material for: Impact of 3D genome organization, guided by cohesin and CTCF looping, on sex-biased chromatin interactions and gene expression in mouse liver
Source: Epigenetics Chromatin. 2020 Jul 17;13:30. doi: 10.1186/s13072-020-00350-y (PMC7368777; doi:10.1186/s13072-020-00350-y)
Supplement: Supplementary file 1 — Additional file 1: Figure S1. Consistency of biological replicates and additional features of sex-biased differential CTCF/cohesin peaks. A-D. Consistency of biological replicate ChIP-seq samples, with merged tracks and annotations as in Figs. 2 and 3. Here, we additionally show all cohesin (n = 3 per sex) and CTCF ChIP-seq replicates (n = 4 per sex). Blue, samples from male mouse liver; red, samples from female mouse liver are shown in red. A. Biological replicates for a male-biased “Lone CTCF” site nearby Mir2136. B. Biological replicates for two female-biased CAC(ΔCoh) peaks nearby 5530601H04Rik. Higher cohesin signal is observed across replicates for both the weaker upstream peak and a strong downstream peak. CTCF signal is not significantly different at either peak. C. Biological replicates for a female-biased CAC peak (ΔCoh AND ΔCTCF) upstream of strong sex-independent peak in an intron of Moh9. D. Biological replicates for several sex-independent CAC peaks of various peak strengths. Replicates are consistent at all sites regardless of peak strength. E. The limited overlap of sex-biased CTCF and sex-biased cohesin binding, seen in Fig. 1B, is not an artifact of the thresholds used for peak filtering. In the data shown here, similar to Fig. 1B, the overlap of sex-biased CTCF and sex-biased cohesin binding sites is limited. Shown are Venn diagrams with the number of overlapping sex-biased cohesin (Left; blue) and CTCF sites (Right; purple) for the following groups (top to bottom): (1) All sex-differential sites output by diffReps without filtering for overlap of a MACS2 ChIP-seq peak; (2) All sex-differential diffReps sites (rather than peaks, which may contain multiple sites) from diffReps that overlap a MACS2 peak for a given factor; (3) All sex-differential hotspots identified by diffReps, which is an alternate method in the diffReps software package to identify differential sites. Specifically, this last approach looks for clusters of differentially co-regul [file 13072_2020_350_MOESM1_ESM.pdf]

Impact of 3D genome organization, guided by cohesin and CTCF looping, on sex-biased chromatin interactions and gene expression in mouse liver

Bryan J. Matthews and David J. Waxman

Department of Biology and Bioinformatics Program, Boston University, Boston, MA 02215

Listing of Supplemental Materials

Additional file 1: contains **Figures S1-S7**

Figure S1: Consistency of biological replicates and additional features of sex-biased differential CTCF/cohesin peaks

Figure S2: Comparison of sex-biased CTCF/cohesin peaks

Figure S3: Tissue conservation features of sex-differential peaks

Figure S4: Additional C8a/C8b screenshot and cohesin depletion effects

Figure S5: Quality control of 4C-seq Samples and A1bg lncRNA analysis

Figure S6: Additional 4C-seq screenshots with replicates

Figure S7: High expression of Nox4 in HCC (both human and mouse model) and other Nox4 screenshots

Additional file 2: Table S3

Additional file 3: Table S1

Additional file 4: Table S4

Additional file 5: Table S2

Supplemental References

Supplemental figure legends

Fig. S1 – Consistency of biological replicates and additional features of sex-biased differential CTCF/cohesin peaks

A-D. Consistency of biological replicate ChIP-seq samples, with merged tracks and annotations as in Figures 2 and 3. Here, we additionally show all cohesin (n=3 per sex) and CTCF ChIP-seq replicates (n=4 per sex). Blue, samples from male mouse liver; red, samples from female mouse liver are shown in red.

A. Biological replicates for a male-biased "Lone CTCF" site nearby *Mir2136*.

B. Biological replicates for two female-biased CAC(ΔCoh) peaks nearby *5530601H04Rik*. Higher cohesin signal is observed across replicates for both the weaker upstream peak and a strong downstream peak. CTCF signal is not significantly different at either peak.

C. Biological replicates for a female-biased CAC peak (ΔCoh AND ΔCTCF) upstream of strong sex-independent peak in an intron of *Moh9*.

D. Biological replicates for several sex-independent CAC peaks of various peak strengths. Replicates are consistent at all sites regardless of peak strength.

E. The limited overlap of sex-biased CTCF and sex-biased cohesin binding, seen in Fig. 1B, is not an artifact of the thresholds used for peak filtering. In the data shown here, similar to Fig. 1B, the overlap of sex-biased CTCF and sex-biased cohesin binding sites is limited. Shown are Venn diagrams with the number of overlapping sex-biased cohesin (*Left*; blue) and CTCF sites (*Right*; purple) for the following groups (top to bottom): (1) All sex-differential sites output by diffReps without filtering for overlap of a MACS2 ChIP-seq peak; (2) All sex-differential diffReps sites (rather than *peaks,* which may contain multiple sites) from diffReps that overlap a MACS2 peak for a given factor; (3) All sex-differential hotspots identified by diffReps, which is an alternate method in the diffReps software package to identify differential sites. Specifically, this last approach looks for clusters of differentially co-regulated sites that might be missed by simple overlap analysis. Overlap for all Venn diagrams is defined as ≥ 1 bp overlapping using bedtools. In some cases, two Rad21 peaks overlap a CTCF peak, or vice versa, and therefore, the number of overlapping cohesin (Rad21) sites does not necessarily equal the number of CTCF sites (hence, two numbers in the Venn overlap).

F. Pie charts showing the class distribution of each sex-biased CTCF/cohesin peak set (from top to bottom): male-biased ΔCohesin peaks, female-biased ΔCohesin peaks, male-biased ΔCTCF peaks, and female-biased ΔCTCF peaks. For each of these four groups, the fraction of peaks at CAC sites is shown in purple while the fraction of peaks at either CNC (for ΔCohesin) or Lone CTCF (for ΔCTCF) is shown in blue. The total number of differential peaks in each group is indicated below each chart. Overall, female-biased sites are comprised of a higher percentage of CAC sites than male-biased sites. Consequently, a larger percentage of male-biased peaks are CNC peaks (for ΔCohesin peaks) and Lone CTCF peak (for ΔCTCF peaks). Peak numbers here differ slightly from Fig. 1B for cohesin differential peaks, but not CTCF, because of our approach to categorizing peaks as CNC or CAC for cohesin peaks (see Methods). For CTCF we defined CAC peaks as genomic regions bound by CTCF that were also bound by cohesin in a majority of individual cohesin replicates (2 or 3 out of a total n=3 per sex). Using the same approach for cohesin, we defined CAC peaks as genomic regions bound by cohesin that were also bound by CTCF in a majority of individual CTCF replicates (3 or 4 out of a total n=4 per sex). If a peak was bound by none or only a minority of replicates for the opposite factor then it was considered Lone CTCF (in the case of CTCF; 0 or 1 cohesin replicates overlapping) or CNC (in the case of cohesin; 0 or 1 CTCF replicates overlapping). As CTCF has n=4 replicates, if a cohesin peak is bound by exactly 2 individual CTCF replicates (of the same sex) then it is not classified and is excluded from downstream analyses. 54 male-biased cohesin peaks overlap 2 male CTCF replicates and 36 female-biased cohesin peaks overlap 2 female CTCF replicates (value of 2 in column H of Table S1B). All overlaps were performed using bedtools with a minimum overlap of 1 bp, and all comparisons were made separately for males and females.

Fig. S1A-S1D

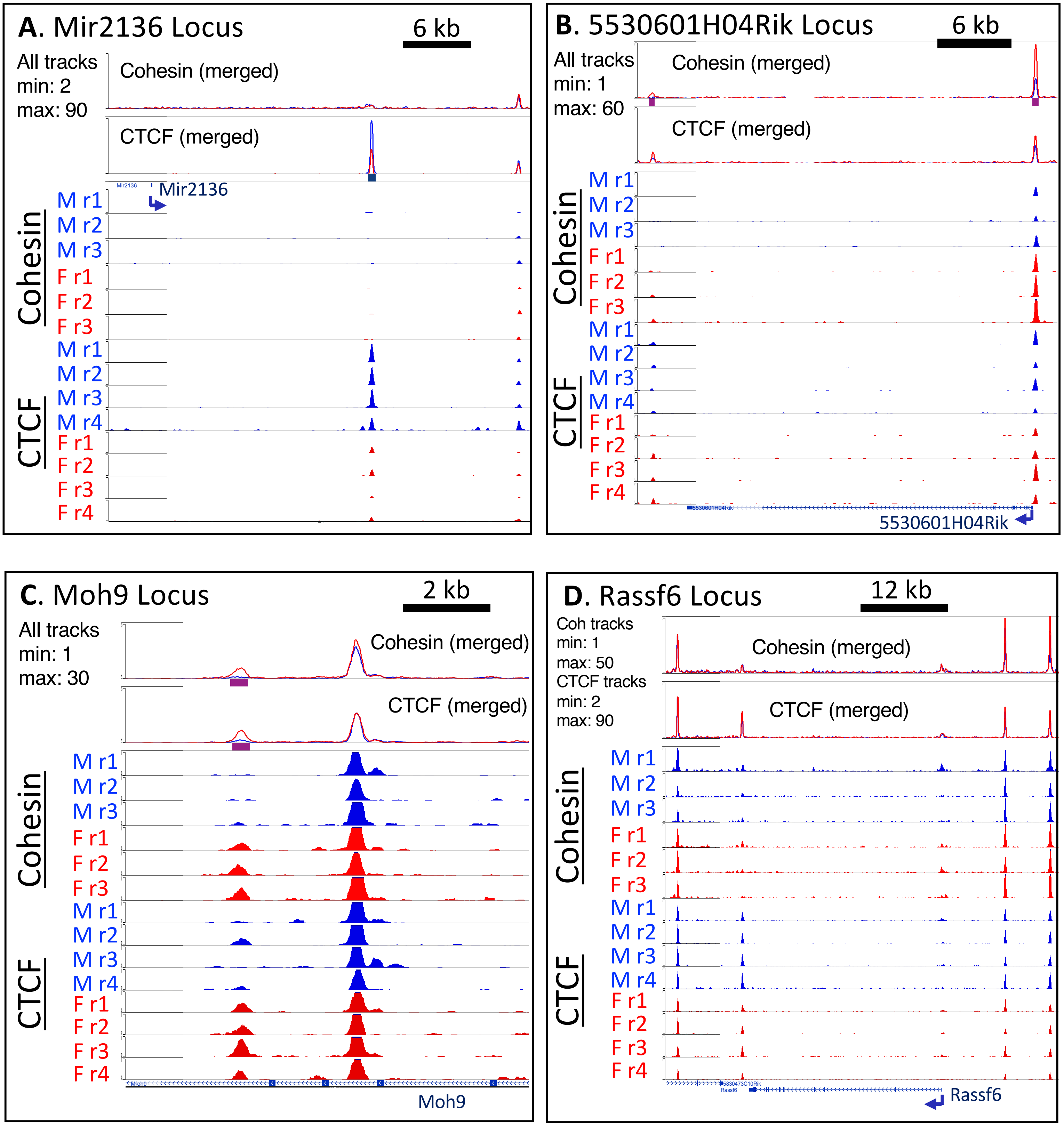
A. Mir2136 Locus
6 kb
All tracks
min: 2
max: 90
Cohesin (merged)
CTCF (merged)
Mir2136
Cohesin
M r1
M r2
M r3
F r1
F r2
F r3
CTCF
M r1
M r2
M r3
M r4
F r1
F r2
F r3
F r4
B. 5530601H04Rik Locus
6 kb
All tracks
min: 1
max: 60
Cohesin (merged)
CTCF (merged)
Cohesin
CTCF
5530601H04Rik
C. Moh9 Locus
2 kb
All tracks
min: 1
max: 30
Cohesin (merged)
CTCF (merged)
Moh9
D. Rassf6 Locus
12 kb
Coh tracks
min: 1
max: 50
CTCF tracks
min: 2
max: 90
Cohesin (merged)
CTCF (merged)
Rassf6

Fig. S1EF

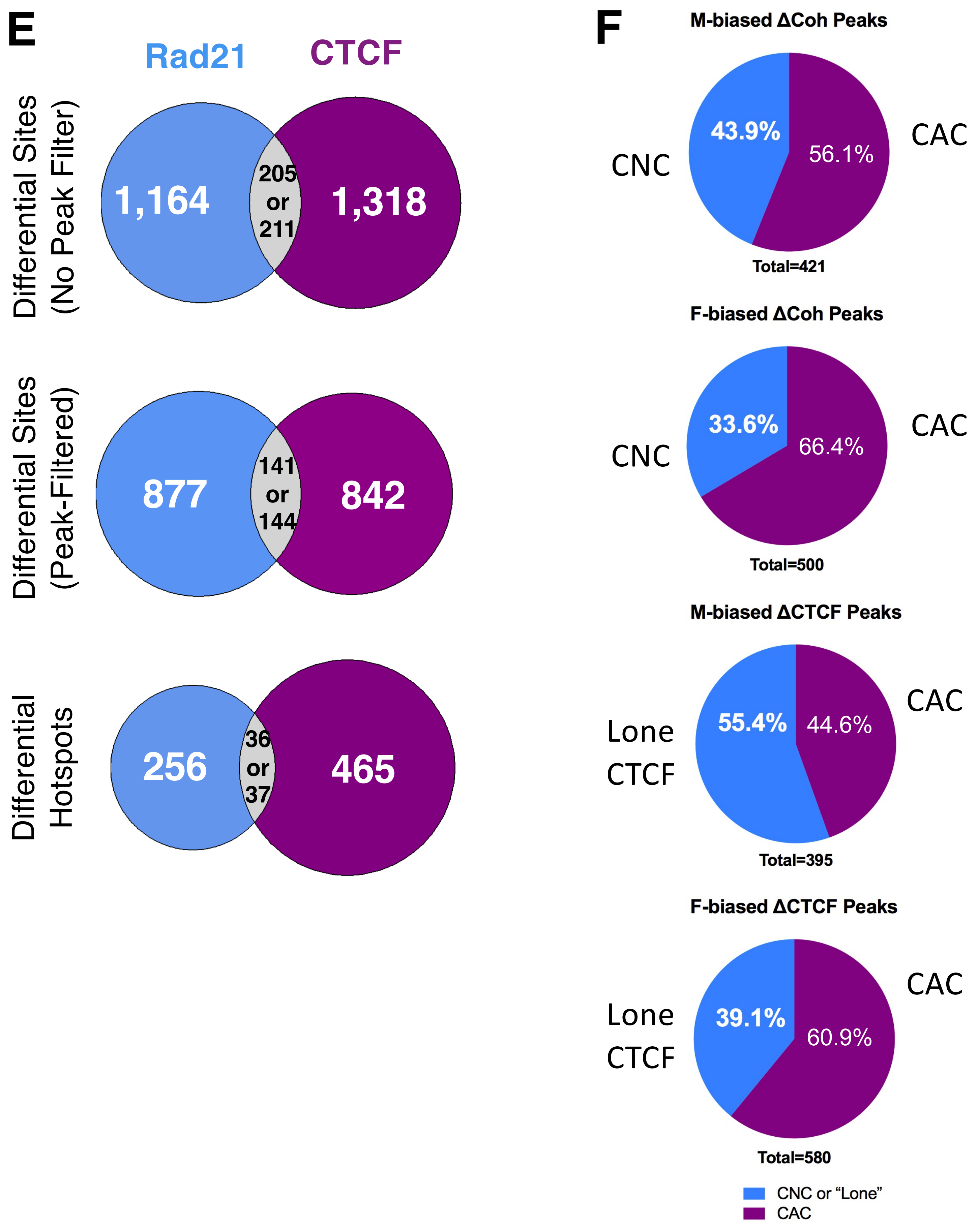
E
Rad21
CTCF
Differential Sites (No Peak Filter)
1,164
205 or 211
1,318
Differential Sites (Peak-Filtered)
877
141 or 144
842
Differential Hotspots
256
36 or 37
465
F
M-biased ΔCoh Peaks
CNC
43.9%
56.1%
CAC
Total=421
F-biased ΔCoh Peaks
CNC
33.6%
66.4%
CAC
Total=500
M-biased ΔCTCF Peaks
Lone CTCF
55.4%
44.6%
CAC
Total=395
F-biased ΔCTCF Peaks
Lone CTCF
39.1%
60.9%
CAC
Total=580
CNC or "Lone"
CAC

Fig. S2 – Comparison of sex-biased CTCF/cohesin peaks

A. Female-biased CTCF and cohesin peaks tend to be stronger than male-biased peaks. Shown here are box plots for ChIP-seq signal for CTCF and cohesin for both ΔCohesin and ΔCTCF peaks. These plots differ from those presented in Fig. 2A, which present normalized ChIP signal for the factor with differential signal (i.e. male and female cohesin ChIP-seq signal for ΔCohesin peaks). In aggregate, CAC peaks with significant sex-biased cohesin binding show the same directionality of sex-bias for CTCF (and vice versa), albeit at a reduced magnitude (see also Fig. 1C). The y-axis shows normalized ChIP-seq signal for the groups indicated along the x-axis. Peaks with male-biased and female-biased cohesin binding (*Left*) and CTCF binding (*Right*) are presented separately. Each group of 4 box plots represents the male and female ChIP-seq signal for cohesin, followed by the corresponding ChIP-seq signals for CTCF for the same set of peaks. Each plot represents all differential peaks for a given sex (male or female) and factor (CTCF or cohesin). These four datasets are further divided by peak type (CAC or CNC for ΔCohesin peaks, and CAC or Lone CTCF for ΔCTCF peaks), as indicated below the x-axis. Peak scores are calculated by average intra-peak ChIP signal, normalized by the total sequence reads per million in peak (RIPM; see Methods).
B. Female-biased CAC peaks contain higher quality CTCF motifs than male-biased CAC peaks [p=0.0212 for CAC(ΔCoh) and p=0.0023 for CAC(ΔCTCF) peaks; M-W t-test], as reflected by the FIMO motif score. This log-likelihood ratio score is a reflection of how close the best intra-peak motif matches the canonical core CTCF motif MA0139.1. There is no significant difference between motif scores for male-biased and female-biased Lone CTCF, or for male-biased and female-biased CNC peaks (p=0.7671 and p=0.1329; M-W t-test). The dashed line at FIMO score = 10 reflects the cutoff used to define the presence or absence of a motif in Fig. S2C.
C. CTCF Motif frequency, based on presence of CTCF motif (MA0139.1) as identified by FIMO, with a minimum score of 10. The y-axis shows the percent of peaks in each group (separately for male-biased, female-biased, and sex-independent) found to have a CTCF motif within the peak region. A larger fraction of female-biased than male-biased CAC peaks was found to contain a CTCF binding motif. In contrast, a larger fraction of male-biased Lone CTCF peaks contain a CTCF motif, despite no significant difference in peak strength between male-biased and female-biased Lone CTCF peaks. A larger fraction of female-biased CNC peaks contain a CTCF motif, however, the vast majority do not contain CTCF motifs, as expected (< 20% for all groups). In all cases, the percent for each group is comparable to a matched set of sex-independent peaks.
D. Proportion of male-biased and female-biased CTCF peaks that have: no CTCF motif (gray), a CTCF motif lacking a CpG dinucleotide (orange), or a CTCF motif containing a CpG dinucleotide (orange). “All” indicates any male-biased or female-biased CTCF peak.
E. Female-biased intra-TAD on Chr19 that contains 12 sex-biased genes, shown in blue and red boxes, some of which are lncRNA genes (ncRNA gene designations, in green). Inset at bottom left of the Figure shows CTCF and cohesin (Rad21) ChIP-seq tracks for male and female mouse liver surrounding the 3’ boundary of the female-biased intra-TAD.
F. Intra-TAD loops and loop anchors are mostly shared between male and female mouse liver. Using a computational intra-TAD loop prediction algorithm [1], we used the cohesin and CTCF ChIP-seq datasets for male and female mouse liver to identify 9,543 intra-TAD loops in male liver [1] and 9,724 loops in female liver, respectively. 87.9% of the intra-TAD loops in male liver were also identified in female liver (*left*), and 93.4% of the male intra-TAD loop anchors are also predicted to be loop anchors in female liver. This finding is consistent with there being a limited number of autosomal CAC peaks with sex differences in CTCF and cohesin binding (53 total) (Table S1C) To account for nested loop structures, shared loops were defined as loops with a reciprocal overlap of 80% or greater between the loops, as implemented in prior studies of CAC-mediated insulating loops [1, 2].

Fig. S2ABCD

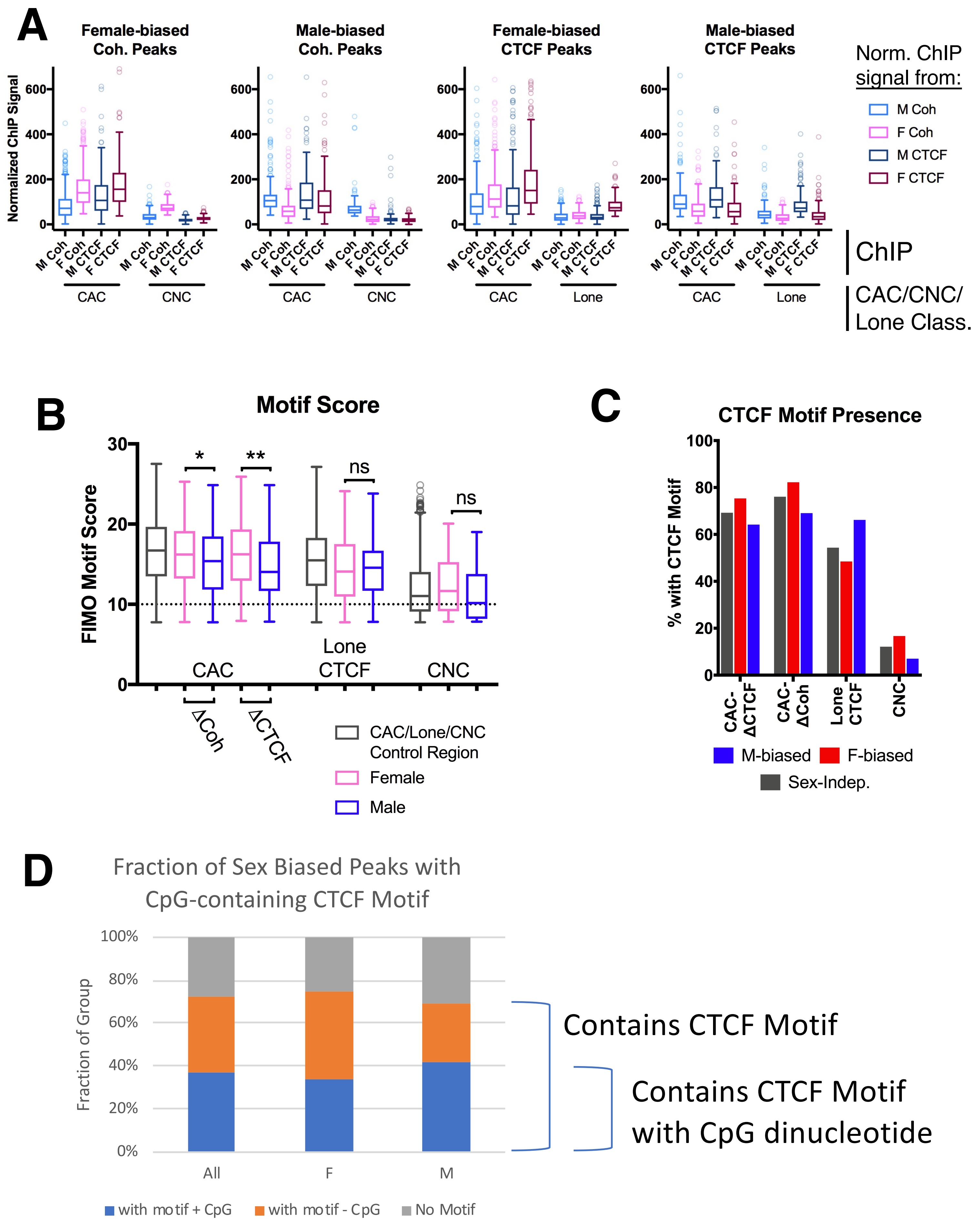
A
Female-biased Coh. Peaks
Male-biased Coh. Peaks
Female-biased CTCF Peaks
Male-biased CTCF Peaks
Normalized ChIP Signal
M Coh
F Coh
M CTCF
F CTCF
CAC
CNC
Lone
Norm. ChIP signal from:
ChIP
CAC/CNC/ Lone Class.
B
Motif Score
FIMO Motif Score
*
**
ns
CAC
Lone CTCF
CNC
ΔCoh
ΔCTCF
CAC/Lone/CNC Control Region
Female
Male
C
CTCF Motif Presence
% with CTCF Motif
CAC-ΔCTCF
CAC-ΔCoh
Lone CTCF
CNC
M-biased
F-biased
Sex-Indep.
D
Fraction of Sex Biased Peaks with CpG-containing CTCF Motif
Fraction of Group
All
F
M
with motif + CpG
with motif - CpG
No Motif
Contains CTCF Motif
Contains CTCF Motif with CpG dinucleotide

E Female-biased intra-TAD containing 12 sex-biased genes

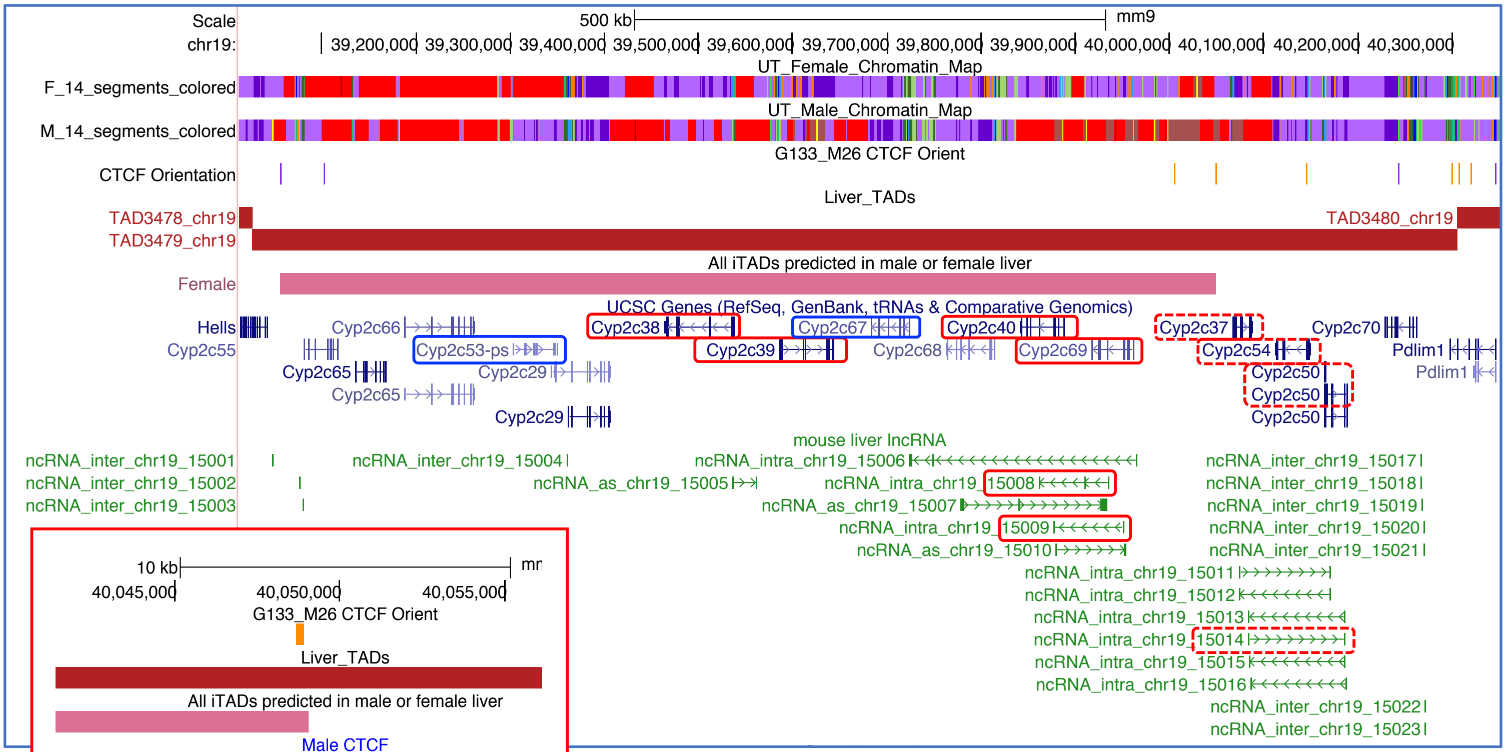

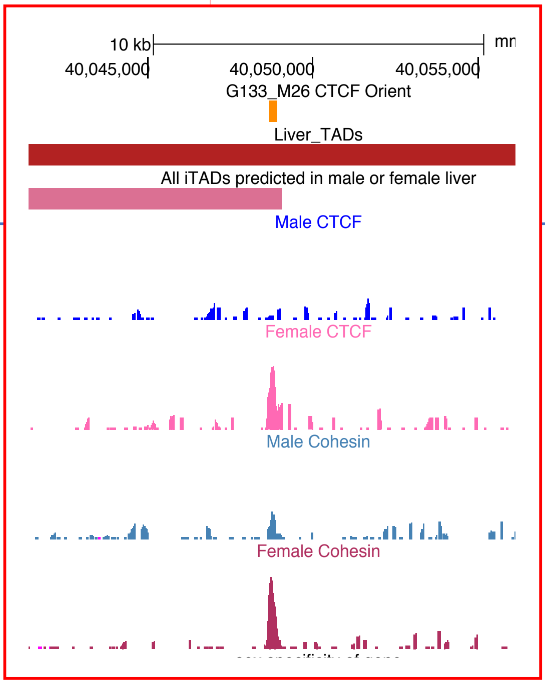

Red rectangles: female-biased gene
Blue rectangles: male-biased gene
Dashed rectangles: sex-biased genes in the TAD but outside the intra-TAD.

Zoom-in view of 3'-end of intra-TAD showing female-biased binding of both CTCT and cohesin (normalized ChIP-seq read wig file).

F

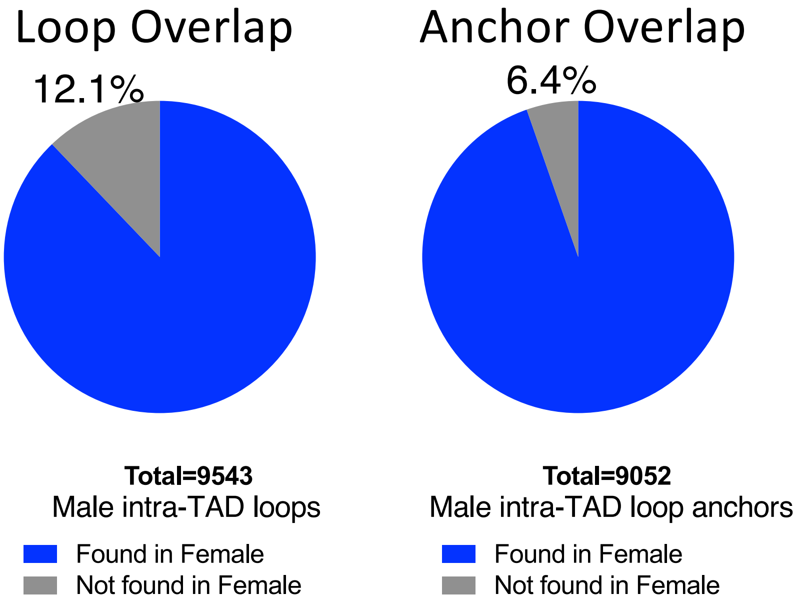

Fig. S3 – Tissue conservation of liver sex-differential CTCF and cohesin peaks in ENCODE mouse consortium datasets.

A. The x-axis indicates the number of male mouse tissues other than liver where CTCF is bound, out of 15 tissues examined by the ENCODE Consortium. A value of 15 indicates tissue-ubiquitous CTCF binding, and a value of 0 indicates liver-specific CTCF binding. The y-axis shows the proportion of male-biased peaks (blue) or female-biased peaks (red) that fall into a given bin. P-values comparing the distribution of tissue-specificity values for CTCF binding between males and females are indicated in the upper *left* corner of each plot (KS t-test). Results show that CAC sites (upper panels) are much less tissue-specific than Lone CTCF and CNC sites (lower panels). Further, female-biased CAC peaks are less tissue-specific than male-biased CAC peaks, while male-biased Lone CTCF peaks are less liver-specific than female-biased peaks of the same class. The greater tissue ubiquity of CTCF binding for female-biased CAC peaks could be due to the fact that female-biased CTCF peaks are stronger and contain higher quality CTCF motifs, insofar as stronger peaks show greater conservation for both sex-differential and all CTCF peaks (see panel B, below); however, male-biased Lone CTCF peaks are not significantly stronger, nor do they contain higher quality motifs than the female-biased Lone CTCF peaks. The apparent difference could be due to the fact that the CTCF ChIP-seq data from the non-liver, non-reproductive tissues examined here was obtained by the ENCODE consortium from male mice [3]. Very few male-biased and female-biased CNC peaks were bound by CTCF in other any other mouse tissues (<20% of the total sex-biased liver CNC sites). This finding provides additional evidence that CNC sites are found at liver-specific *cis* regulatory elements, and that these sites do not act as insulators in other non-liver tissues (i.e., CTCF binding is lost in liver or gained in some other tissue).
B. There is a strong association between CTCF peak strength and tissue conservation of CTCF binding, which likely explains the modestly higher tissue conservation of female-biased CTCF and cohesin peaks seen in panel A. Shown on the y-axis are reads-in-peaks normalized ChIP-seq data for all CTCF peaks, male-biased CTCF peaks, and female-biased CTCF peaks. These are grouped according to the number of non-liver ENCODE tissues with a CTCF peak, where 0 indicates a peak is liver-specific and 15 means all male mouse tissues with ENCODE datasets have CTCF bound at that position, as in panel A.
C. The tissue specificity of neighboring genes varies significantly with the class of CTCF/cohesin binding site. Shown are Tau values, where a value close to 1 indicates the pattern of expression across mouse ENCODE RNA-seq datasets is highly tissue-specific, and where Tau values less than ~0.3 indicate housekeeping genes. Nearest genes (within 20 kb) were defined based on distance to the TSS, and only liver-expressed genes were considered (FPKM > 1). Tau values were calculated based on the average of two replicates from all tissues except testis, using expression data generated by the ENCODE consortium. Both female-biased and male-biased CNC sites are near genes that generally are more tissue-specific than liver-expressed genes. In addition, genes near female-biased CNC sites are significantly different than genes near similarly sex-biased CACs (p=0.007; M-W test). This difference is not a reflection of the male-biased or female-biased CAC group used in the comparison, insofar as genes near female-biased CNCs are significantly more liver-specific than genes near male-biased CAC sites (p=0.0171; M-W test), while the opposite comparison for male-biased CNCs vs female-biased CACs is still not significant (p=0.07; M-W test). For these analyses, liver-expressed genes are defined by a liver expression value of FPKM > 1 (8,810 genes in total) and mapping was based on the closest TSS within 20 kb of a peak.

Fig. S3

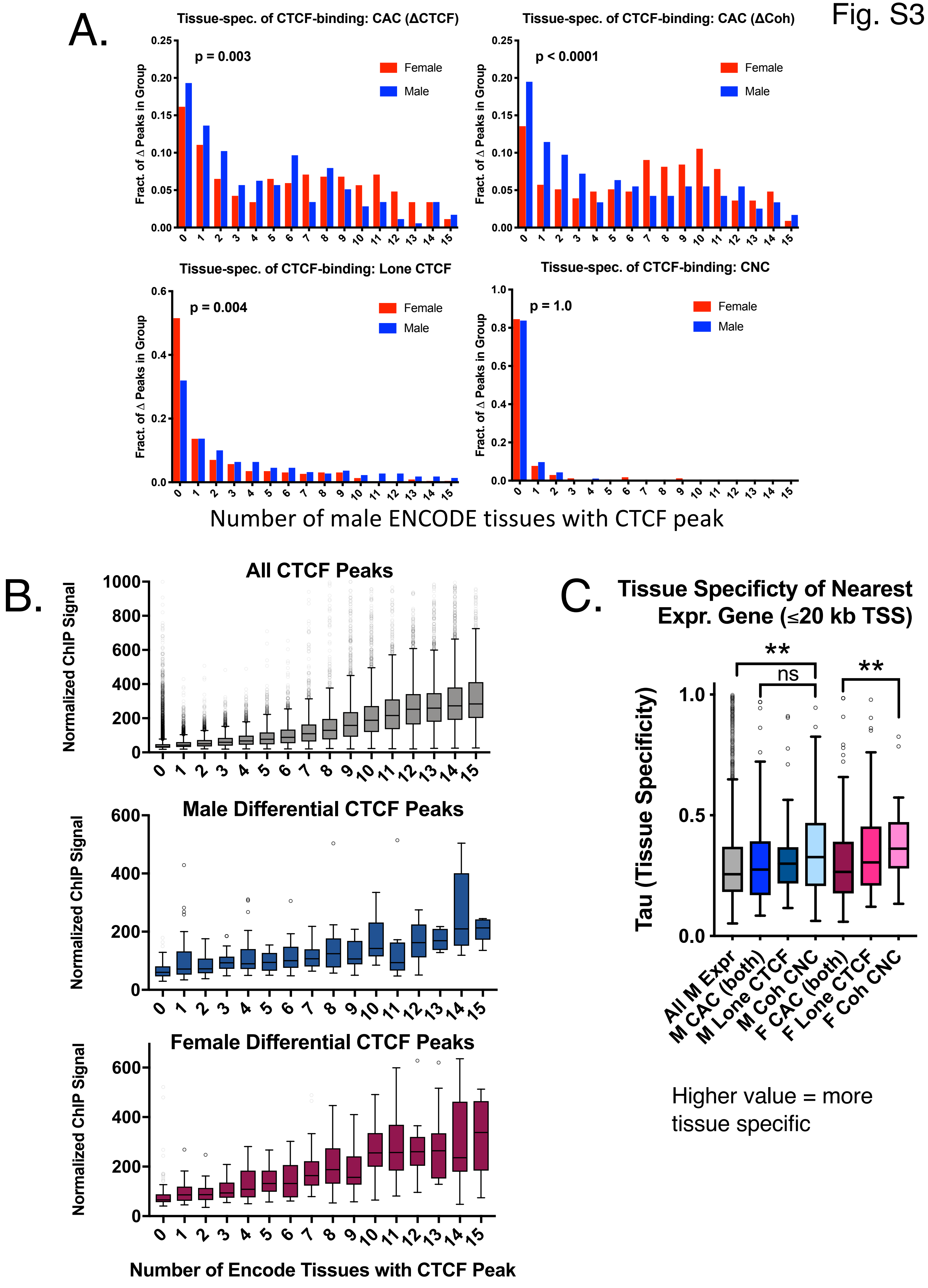
A.
Tissue-spec. of CTCF-binding: CAC (ΔCTCF)
p = 0.003
Tissue-spec. of CTCF-binding: CAC (ΔCoh)
p < 0.0001
Tissue-spec. of CTCF-binding: Lone CTCF
p = 0.004
Tissue-spec. of CTCF-binding: CNC
p = 1.0
Female
Male
Fract. of Δ Peaks in Group
Number of male ENCODE tissues with CTCF peak
B.
All CTCF Peaks
Male Differential CTCF Peaks
Female Differential CTCF Peaks
Normalized ChIP Signal
Number of Encode Tissues with CTCF Peak
C.
Tissue Specificty of Nearest Expr. Gene (≤20 kb TSS)
**
ns
**
Tau (Tissue Specificity)
All M Expr
M CAC (both)
M Lone CTCF
M Coh CNC
F CAC (both)
F Lone CTCF
F Coh CNC
Higher value = more tissue specific

Fig. S4 – Screenshot of TAD containing C8a/C8b, and cohesin depletion effects

A. Shown is a screenshot with proposed model linking the distal male-biased enhancers and component complement genes *C8a* and *C8b* within a single TAD on mouse chromosome 4. This screenshot spans chr4:102960671-104603975. The tracks, normalization, and annotations are as described in Fig. 3.
B. For the proximally-regulated male-biased genes shown in Fig. 3B (*Nat8* and *Cml5*), depletion of cohesin does not significantly impact gene expression.

Fig. S4

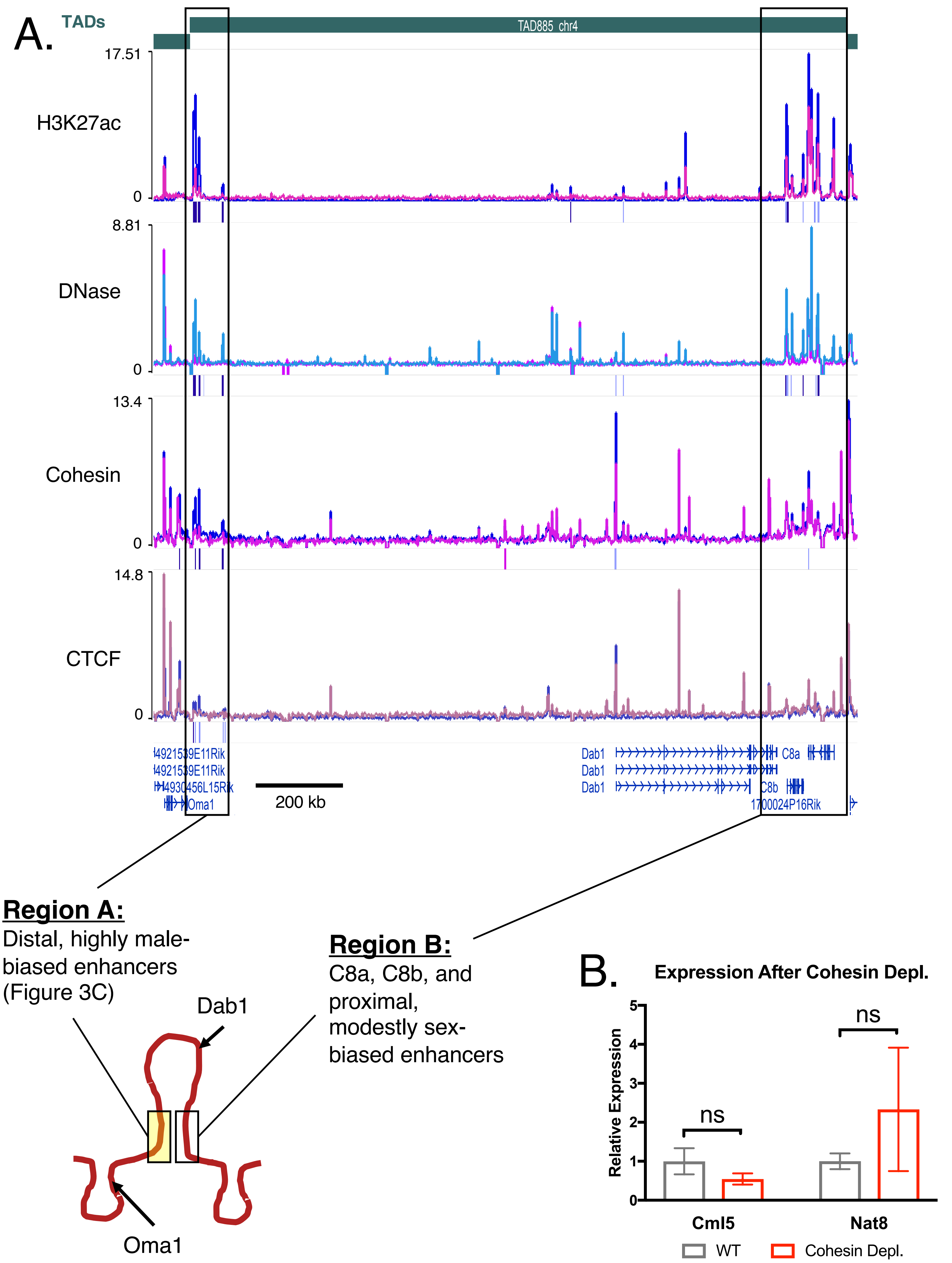
A.
TADs
TAD885_chr4
17.51
H3K27ac
0
8.81
DNase
0
13.4
Cohesin
0
14.8
CTCF
0
4921539E11Rik
4921539E11Rik
4930456L15Rik
Oma1
200 kb
Dab1
Dab1
Dab1
C8a
C8b
1700024P16Rik
Region A:
Distal, highly male-biased enhancers (Figure 3C)
Region B:
C8a, C8b, and proximal, modestly sex-biased enhancers
Dab1
Oma1
B.
Expression After Cohesin Depl.
Relative Expression
5
4
3
2
1
0
ns
ns
Cml5
Nat8
WT
Cohesin Depl.

Fig. S5 – Quality control of 4C-seq library and expression data for A1bg region

A. Agarose gel analysis for quality control of ligated, digested, and re-ligated 4C samples. Lane (i) analyzes a sample after proximity ligation, lane (ii) shows the sample after digestion with the restriction enzyme Csp6i, and lane (iii) shows the sample after self-circularization ligation. Lane (iii) represents the final material used as input for inverse PCR with viewpoint-specific primers (Table S3A). DNA fragment sizes (in kb) are marked on the *left* of the gel.
B. Agarose gel analysis for quality control of final 4C-seq libraries after the inverse PCR step. A diverse mixture of PCR products is present, as indicated by a smear on the gel, with sizes primarily below ~1 kb, which indicate a high-quality library and which allows for efficient sequencing. DNA fragment sizes (in kb) are marked on the *right* of the gel.
C. Shown is RNA-seq expression data for *A1bg* and 12 mono-exonic lncRNAs (see Fig. 4A), obtained in six separate RNA-seq datasets from CD-1 mouse liver, and one RNA-seq dataset from C57/Bl6 mouse liver. The first two columns indicate the maximum expression (in FPKM) for male and female liver across these datasets. Following this from left to right, the columns indicate the mean expression level of each gene in female liver (FPKM values) for: Total PolyA+ unstranded RNA-seq [sequencing series G83; [4]], Total PolyA+ unstranded RNA-seq [sequencing series G85; [5]], Total PolyA+ stranded RNA-seq [sequencing series G118; [4]], Nuclear PolyA+ stranded RNA-seq [sequencing series G119; [4]], Total Ribosomal RNA-depleted stranded RNA-seq [sequencing series G118; [4]], Nuclear Ribosomal RNA-depleted stranded RNA-seq [sequencing series G119; [4]], and Total Ribosomal RNA-depleted stranded RNA-seq [from C57Bl6/J, all others CD-1; [6]]. The final columns indicate the nuclear enrichment for PolyA+ RNA datasets and for Ribosomal RNA-depleted RNA-seq datasets (linear scale). Specifically, for PolyA+ datasets, this is the FPKM value in data column 6 ("Nuclear Poly A Strnd") divided by the FPKM value in data column 5 ("Total Poly A Strnd"). Similarly, the final column is calculated for Ribosomal RNA-Depleted Nuclear versus total RNA-seq expression (FPKM in data column 8 "Nuclear RiboM Strnd" dividied by the FPKM value in data column 7 "Total RiboM Strnd").
D. Shown are the log2 male/female expression ratios for *A1bg* and the 12 mono-exonic lncRNAs (Fig. 4A) for the sven RNA-seq datasets described in panel C. Fold change is calculated by EdgeR for all datasets and the order of datasets is the same as in panel C. The final column indicates the number of datasets in which the sex differences indicated are significant (FDR < 0 .05; EdgeR).

Fig. S5

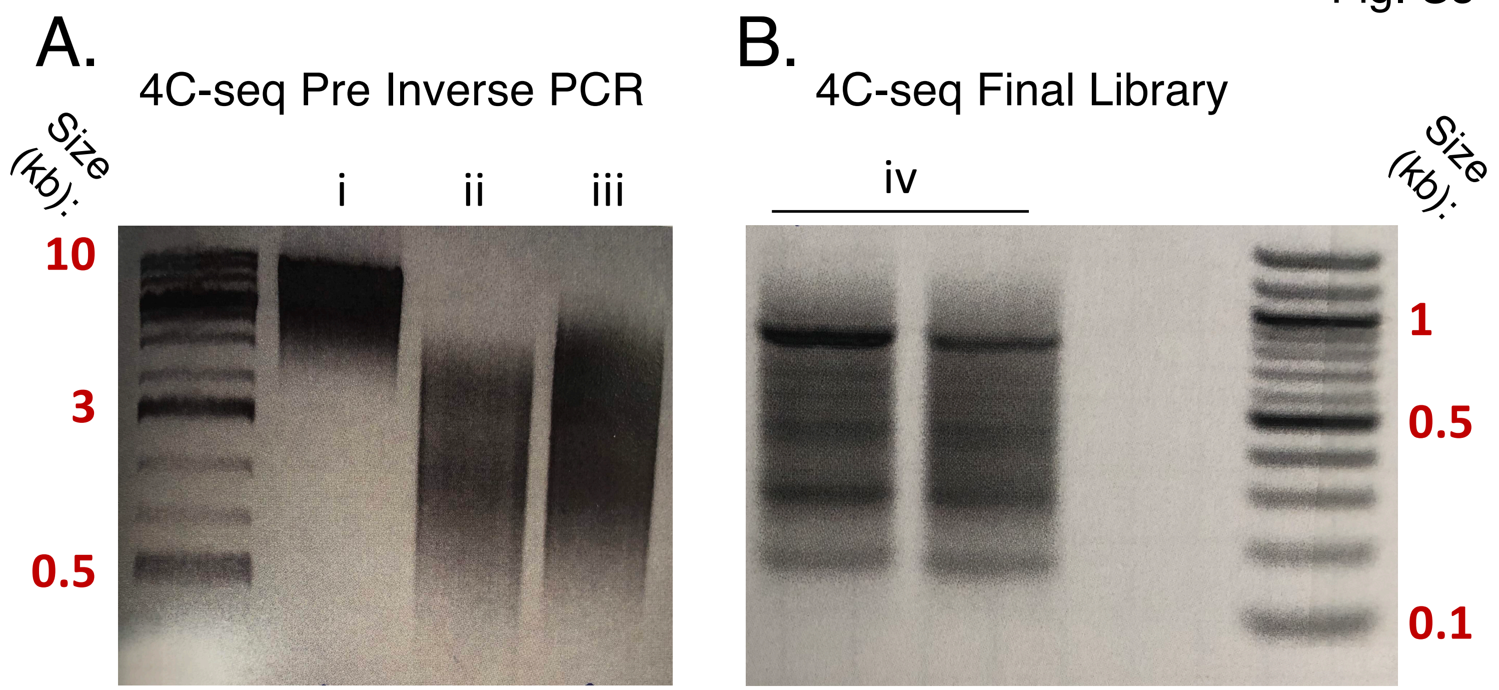

C. **Expression Across Datasets (F FPKM)**

	Max FPKM Male	Max FPKM Female	Total PolyA Unstr F (G83)	PolyA Unstr F (G85)	Total PolyA Strnd F	Nuclear PolyA Strnd F	Total RiboM Strnd F	Nuclear RiboM Strnd F	B6 Total RiboM Strnd F	Nuclear Enrichment	
										Nuclear Enrichment (PolyA)	Nuclear Enrichment (RiboM)
ncRNA_inter_chr15_12590	0.01	2.71	0.62	1.30	0.54	2.57	0.67	2.71	0.13	4.8	4.1
ncRNA_inter_chr15_12591	0.01	2.11	0.35	0.97	0.50	1.15	0.53	2.11	0.18	2.3	4.0
ncRNA_inter_chr15_12592	0	0.99	0.25	0.35	0.06	0.22	0.29	0.99	0.07	3.6	3.5
ncRNA_inter_chr15_12593	0	1.80	0.06	0.19	0.09	1.06	0.31	1.80	0.12	11.4	5.9
ncRNA_inter_chr15_12595	0.01	1.13	0.01	0.01	0.01	0.33	0.23	1.13	0.03	38.8	5.0
ncRNA_inter_chr15_12596	0.02	1.77	0.02	0	0.03	0.24	0.15	1.77	0.07	9.3	11.8
ncRNA_inter_chr15_12597	0	0.91	0.01	0.03	0.02	0.34	0.09	0.91	0.04	15.0	9.9
ncRNA_inter_chr15_12598	0	0.87	0.06	0.00	0.07	0.68	0.11	0.87	0.02	9.2	8.1
ncRNA_inter_chr15_12599	0.02	0.81	0.02	0.04	0.03	0.81	0.09	0.73	0.01	27.9	7.8
ncRNA_inter_chr15_12600	0	0.37	0	0	0.02	0	0.03	0.37	0	0.0	11.8
ncRNA_inter_chr15_12601	0.01	0.31	0	0	0	0.06	0.00	0.31	0	inf	inf
ncRNA_inter_chr15_12602	0	0.34	0	0	0.01	0.03	0.02	0.34	0	2.6	15.1
A1bg	4.35	1,422.30	846.40	1,422.30	1,238.83	382.97	250.99	37.52	522.16	0.31	0.15

Highlighting = relative within column for lncRNAs only (excluding A1bg)
NE = (Female Nuclear FPKM / Female Total FPKM)

D. **FC of Significant Sex Bias (log2; M/F)**

	Log2 FC (M/F) from EdgeR							
	PolyA Unstr F (G83)	PolyA Unstr F (G85)	Total PolyA Strnd F	Nuclear PolyA Strnd F	Total RiboM Strnd F	Nuclear RiboM Strnd F	B6 Total RiboM Strnd F	# of datasets sign. in
ncRNA_inter_chr15_12590	-6.519	-6.870	-8.203	-10.232	-7.557	-9.850	-4.453	7
ncRNA_inter_chr15_12591	-4.937	-8.111	-5.436	-8.313	-6.344	-8.715	-4.078	7
ncRNA_inter_chr15_12592	-6.358	-6.359	ns	-5.682	-5.331	-7.359	ns	5
ncRNA_inter_chr15_12593	ns	ns	ns	-7.893	-5.347	-8.189	-3.330	4
ncRNA_inter_chr15_12595	ns	ns	ns	-6.804	-5.586	-6.170	ns	3
ncRNA_inter_chr15_12596	ns	ns	ns	-5.284	ns	-7.703	ns	2
ncRNA_inter_chr15_12597	ns	ns	ns	-6.741	ns	-7.682	ns	2
ncRNA_inter_chr15_12598	ns	ns	ns	-6.147	ns	-6.023	ns	2
ncRNA_inter_chr15_12599	ns	ns	ns	-7.073	ns	-6.459	ns	2
ncRNA_inter_chr15_12600	ns	ns	ns	ns	ns	-5.199	ns	1
ncRNA_inter_chr15_12601	ns	ns	ns	ns	ns	-5.464	ns	1
ncRNA_inter_chr15_12602	ns	ns	ns	ns	ns	-5.388	ns	1
A1bg	-7.620	-10.348	-8.914	-9.734	-7.278	-7.964	-12.38	7

Highlighting = relative within table for sex bias and relative within column for "# of datasets sign. In"
"# of datasets..." = the number of RNA-seq datasets (left) in which significant sex bias is observed (max of 7)

Fig. S6 – 4C-seq data tracks for individual male and female mouse livers

Shown are 4C-seq data for the same genomic regions presented in Fig. 4, but showing the individual 4C-seq data for each of 3 male liver and 3 female liver biological replicates per viewpoint. The gene tracks and sex-biased sites are as described in Fig. 4. These bed file tracks are as follows (from top to bottom): sex-biased H3K27ac peaks, sex-biased DHS, sex-biased cohesin peaks, and sex-biased CTCF peaks. Protein coding genes, sex-biased lncRNA genes, and intra-TAD loops are also shown, where present.
A. All six biological replicates for the *A1bg* enhancer viewpoint (chr15:60733512-60954051).
B. All six biological replicates for the *Gm4794* enhancer viewpoint (chr10:33418446-33680888).
C. All six biological replicates for the *C9* promoter viewpoint (chr15:6147917-6461799).
D. All six biological replicates for the *Nudt7* enhancer viewpoint (chr8:116592444-116707613).
E. Distal enhancer regions from panel C with bidirectional eRNA loci and YY1 binding sites indicated in relation to male-biased enhancers (chr15:6,164,877-6,184,670).

Fig. S6A-S6D

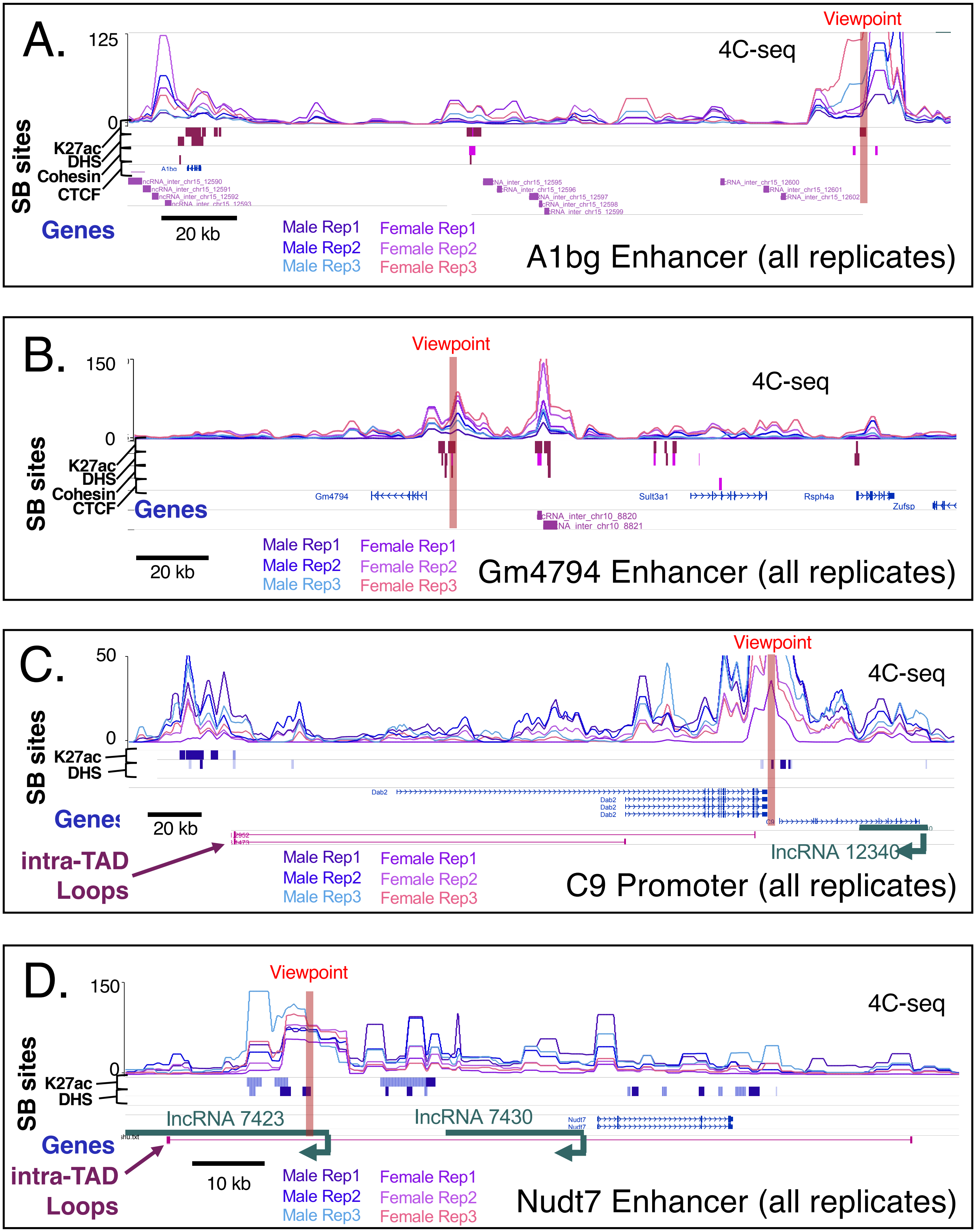
A.
125
0
Viewpoint
4C-seq
SB sites
K27ac
DHS
Cohesin
CTCF
Genes
20 kb
Male Rep1
Male Rep2
Male Rep3
Female Rep1
Female Rep2
Female Rep3
A1bg Enhancer (all replicates)
B.
150
0
Viewpoint
4C-seq
SB sites
K27ac
DHS
Cohesin
CTCF
Genes
Gm4794
Sult3a1
Rsph4a
Zufsp
20 kb
Male Rep1
Male Rep2
Male Rep3
Female Rep1
Female Rep2
Female Rep3
Gm4794 Enhancer (all replicates)
C.
50
0
Viewpoint
4C-seq
SB sites
K27ac
DHS
Genes
Dab2
20 kb
intra-TAD
Loops
lncRNA 12340
Male Rep1
Male Rep2
Male Rep3
Female Rep1
Female Rep2
Female Rep3
C9 Promoter (all replicates)
D.
150
0
Viewpoint
4C-seq
SB sites
K27ac
DHS
lncRNA 7423
lncRNA 7430
Nudt7
Genes
intra-TAD
Loops
10 kb
Male Rep1
Male Rep2
Male Rep3
Female Rep1
Female Rep2
Female Rep3
Nudt7 Enhancer (all replicates)

Fig. S6E

E.

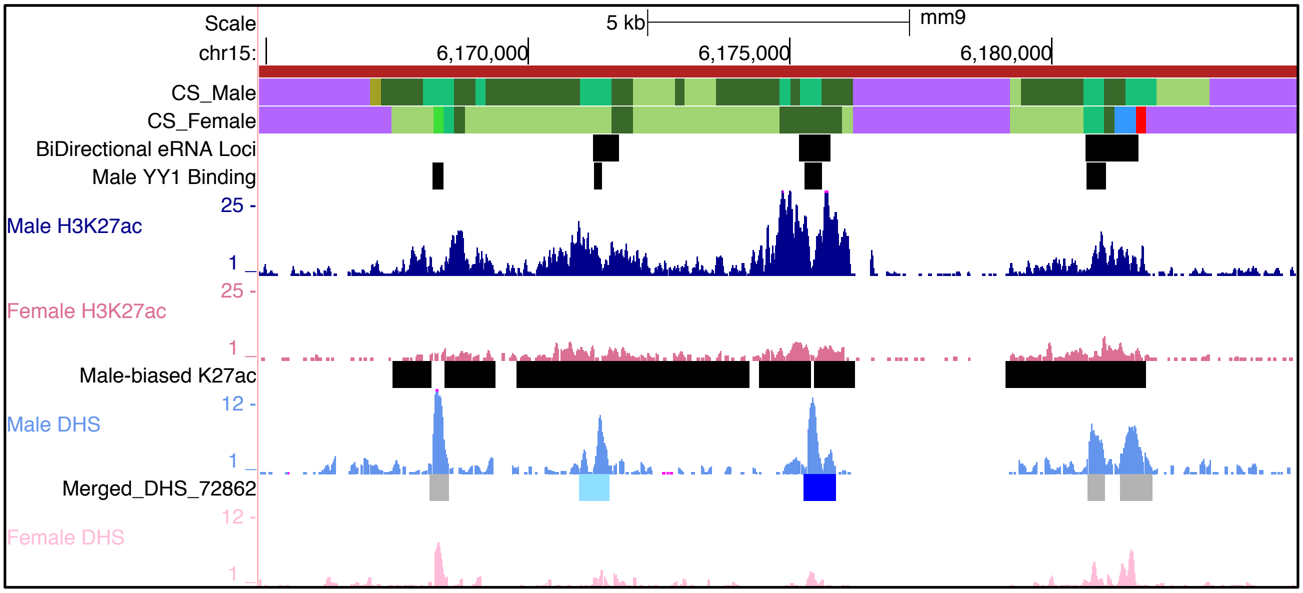

Scale
5 kb
mm9
chr15:
6,170,000
6,175,000
6,180,000
CS_Male
CS_Female
BiDirectional eRNA Loci
Male YY1 Binding
25 -
Male H3K27ac
1 _
25 -
Female H3K27ac
1 _
Male-biased K27ac
12 -
Male DHS
1 _
Merged_DHS_72862
12 -
Female DHS
1 _

Fig. S7 – High expression of *Nox4* in hepatocellular carcinoma and 4C-seq biological replicates

A. *Nox4* is highly up regulated in tumor relative to normal healthy tissue of mice that spontaneously develop tumors (mouse strain C3H; [7]) (p=0.0006, M-W t-test).
B. In human patient samples, *Nox4* is consistently up regulated in tumor tissue relative to normal controls, including in hepatocellular carcinoma (marked by thick black line). Only data for cancer types with matched primary tumor and normal tissue controls are shown, with *Nox4* showing significant up regulation in tumors for 14 of 18 matched tissue pairs. Expression datasets are from The Cancer Genome Atlas (TCGA) and were analyzed by the Tumor IMmune Estimation Resource (TIMER) webtool (https://cistrome.shinyapps.io/timer/) with default parameters [8]. The significance of comparisons between Normal and Tumor tissue was calculated by Wilcoxon test and is indicated on the chart as: 0 ≤ *** < 0.001 ≤ ** < 0.01 ≤ * < 0.05. For example, the difference between liver tumor versus normal tissue (p = 1.35E-25) is indicated as ***.
C. Chromatin state key for *top* browser track in Fig. 5A, which is based on chromatin states in both male and female mouse liver, which were determined for the entire genome based on a 14-state model of chromatin states developed in [9].
D. Male and female 4C-seq biological replicates for the *Nox4* enhancer-1 viewpoint (chr7:94248242-94726358). Tracks are as described in Fig. S6. "SB Peaks" indicate the male-biased (blue) and female-biased (pink) for the indicated factor or assay and a darker color indicates a more stringently-defined sex-biased region (cutoffs differ for each factor; see Methods). These tracks, from top to bottom, are as follows: H3K27ac ChIP-seq, DNase-seq, Rad21 ChIP-seq, and CTCF ChIP-seq and are as described in Fig. 3 and Fig. 4. A complete listing of all such peaks is provided in Tables 2D and 2E (K27ac ChIP-seq and DNase-seq) and in Tables 1D, and 1E (CTCF and cohesin ChIP-seq peaks).
E. Male and female 4C-seq biological replicates for the *Nox4* enhancer-2 viewpoint (chr7:94248242-94726358). Tracks are as described in Fig. S6 and in panel D of this Figure.
F. Three separate models of regulatory domain prediction support the model that *Nox4* enhancer-1 viewpoint and enhancer-2 viewpoint (VP1, VP2) do not interact. The position of enhancer-1 viewpoint is indicated by a red arrow, and the position of enhancer-2 viewpoint is indicated by a green arrow. From *top* to *bottom*: Enhancer Promoter Units (EPUs) are domains based on the pan-tissue correlation of ChIP-seq signal at enhancer and promoters [3]; intra-TAD loops are computationally predicted based on CTCF and cohesin ChIP-seq data for male mouse liver [1]; cohesin ChIA-PET loops show cohesin-anchored interactions from mouse embryonic stem cells [2].

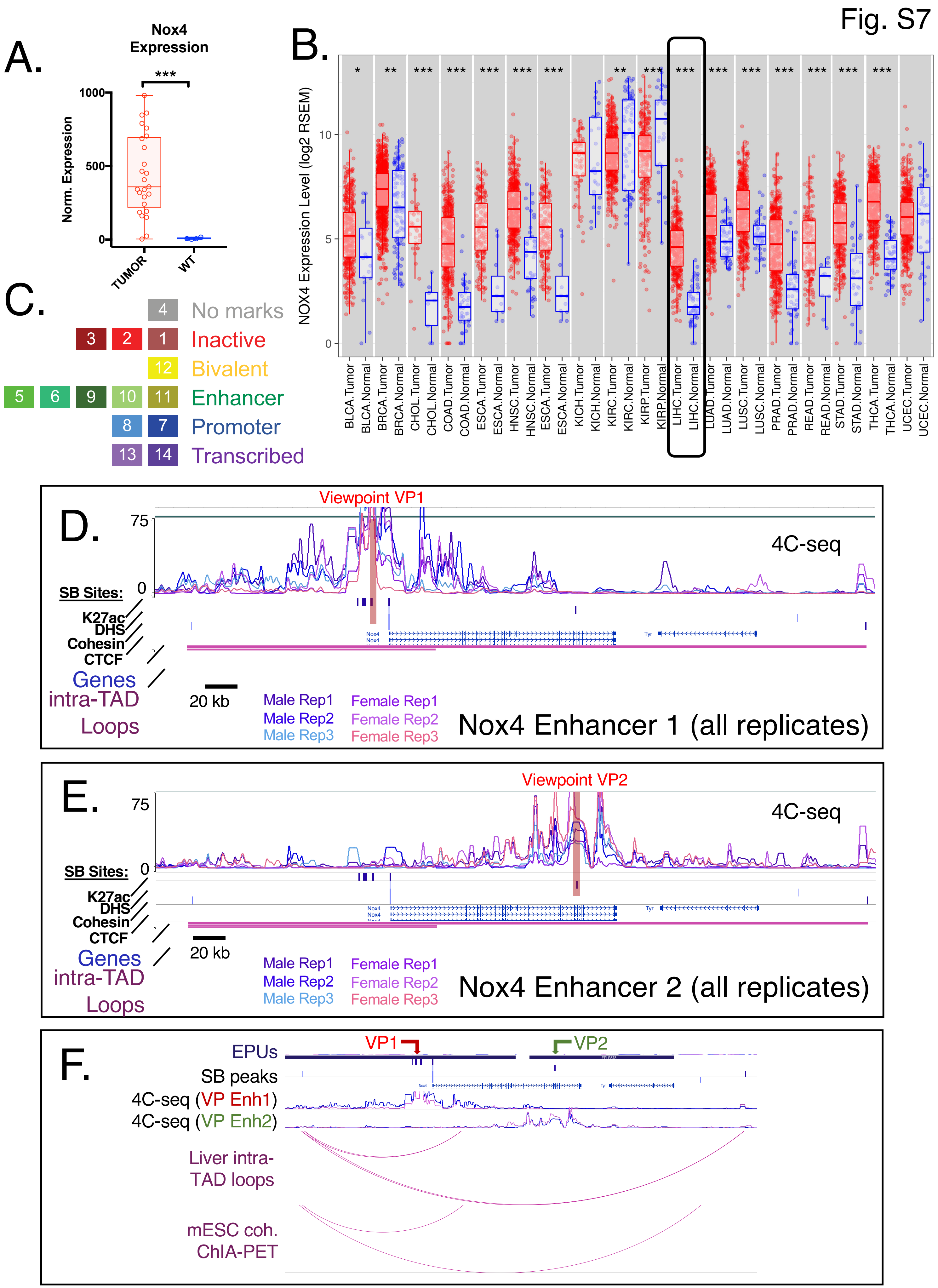
Fig. S7
A.
Nox4 Expression
***
Norm. Expression
1000
500
0
TUMOR
WT
B.
NOX4 Expression Level (log2 RSEM)
BLCA.Tumor
BLCA.Normal
BRCA.Tumor
BRCA.Normal
CHOL.Tumor
CHOL.Normal
COAD.Tumor
COAD.Normal
ESCA.Tumor
ESCA.Normal
HNSC.Tumor
HNSC.Normal
ESCA.Tumor
ESCA.Normal
KICH.Tumor
KICH.Normal
KIRC.Tumor
KIRC.Normal
KIRP.Tumor
KIRP.Normal
LIHC.Tumor
LIHC.Normal
LUAD.Tumor
LUAD.Normal
LUSC.Tumor
LUSC.Normal
PRAD.Tumor
PRAD.Normal
READ.Tumor
READ.Normal
STAD.Tumor
STAD.Normal
THCA.Tumor
THCA.Normal
UCEC.Tumor
UCEC.Normal
C.
4 No marks
3 2 1 Inactive
12 Bivalent
5 6 9 10 11 Enhancer
8 7 Promoter
13 14 Transcribed
D.
Viewpoint VP1
75
0
4C-seq
SB Sites:
K27ac
DHS
Cohesin
CTCF
Genes
intra-TAD
Loops
20 kb
Male Rep1
Male Rep2
Male Rep3
Female Rep1
Female Rep2
Female Rep3
Nox4 Enhancer 1 (all replicates)
E.
Viewpoint VP2
75
0
4C-seq
SB Sites:
K27ac
DHS
Cohesin
CTCF
Genes
intra-TAD
Loops
20 kb
Male Rep1
Male Rep2
Male Rep3
Female Rep1
Female Rep2
Female Rep3
Nox4 Enhancer 2 (all replicates)
F.
EPUs
VP1
VP2
SB peaks
4C-seq (VP Enh1)
4C-seq (VP Enh2)
Liver intra-TAD loops
mESC coh. ChIA-PET

References for Supplemental Figure legends

1. Matthews BJ, Waxman DJ: **Computational prediction of CTCF/cohesin-based intra-TAD loops that insulate chromatin contacts and gene expression in mouse liver**. *eLife* 2018, **7**.
2. Dowen JM, Fan ZP, Hnisz D, Ren G, Abraham BJ, Zhang LN, Weintraub AS, Schuijers J, Lee TI, Zhao K *et al*: **Control of cell identity genes occurs in insulated neighborhoods in mammalian chromosomes**. *Cell* 2014, **159**(2):374-387.
3. Shen Y, Yue F, McCleary DF, Ye Z, Edsall L, Kuan S, Wagner U, Dixon J, Lee L, Lobanenkov VV *et al*: **A map of the cis-regulatory sequences in the mouse genome**. *Nature* 2012, **488**(7409):116-120.
4. Lau-Corona D, Suvorov A, Waxman DJ: **Feminization of Male Mouse Liver by Persistent Growth Hormone Stimulation: Activation of Sex-Biased Transcriptional Networks and Dynamic Changes in Chromatin States**. *Mol Cell Biol* 2017, **37**(19).
5. Connerney J, Lau-Corona D, Rampersaud A, Waxman DJ: **Activation of Male Liver Chromatin Accessibility and STAT5-Dependent Gene Transcription by Plasma Growth Hormone Pulses**. *Endocrinology* 2017, **158**(5):1386-1405.
6. Lowe R, Gemma C, Rakyan VK, Holland ML: **Sexually dimorphic gene expression emerges with embryonic genome activation and is dynamic throughout development**. *BMC Genomics* 2015, **16**(1):295.
7. Aitken SJ, Ibarra-Soria X, Kentepozidou E, Flicek P, Feig C, Marioni JC, Odom DT: **CTCF maintains regulatory homeostasis of cancer pathways**. *Genome Biol* 2018, **19**(1):106.
8. Li T, Fan J, Wang B, Traugh N, Chen Q, Liu JS, Li B, Liu XS: **TIMER: A Web Server for Comprehensive Analysis of Tumor-Infiltrating Immune Cells**. *Cancer Res* 2017, **77**(21):e108-e110.
9. Sugathan A, Waxman DJ: **Genome-wide analysis of chromatin states reveals distinct mechanisms of sex-dependent gene regulation in male and female mouse liver**. *Mol Cell Biol* 2013, **33**(18):3594-3610.
